# No Place

**Published:** 1874-07

**Authors:** 


### NO PLACE.

THERE is always a place for every one and one more, if the person who wants a place will adapt himself to the circumstances of the case, and is fit for the position. Most young men want more than their services are worth to the person employing them. If you can't get the place you want, instead of waiting for a vacancy, don't waste a day, not an hour, because by so doing you are wasting your resources; better take a position which only pays board—discharge promptly, well, and faithfully as is possible for you, the duties of that position, and one of two things will be the inevitable result, almost always—your employer will either increase your wages for that work or will put you up higher. All that you have to do is to perform your duties so well that he will feel he cannot conveniently do without you; for wages take what you can get, and with promptitude, fidelity, thoroughness and integrity, you will, sooner or later, command your price. Be willing to lend a hand to anything connected with the business; show as much interest in it as if it were your own, and in most cases you will become a partner in the concern. I know a man in New York who, as a boy, swept out the bank, and now he is its honored, capable and trusted cashier, with ten thousand a year. I know another who lighted the fires of a drug store at fourteen; the other day he sold his Fifth avenue mansion for a hundred and thirty thousand dollars. The young men who do not keep their places are those who smoke segars, sport canes, begin to watch the minute hand half an hour before the closing of the store, and would think it a great hardship to be kept in an emergency half an hour later; who take up every cent of their salary the day it is due; who call for "something to drink" at their lunches, patronize theatres, and loiter about public places on Sunday.

